# Helminth Parasites Alter Protection against *Plasmodium* Infection

**DOI:** 10.1155/2014/913696

**Published:** 2014-09-08

**Authors:** Víctor H. Salazar-Castañon, Martha Legorreta-Herrera, Miriam Rodriguez-Sosa

**Affiliations:** ^1^Unidad de Biomedicina, Facultad de Estudios Superiores-Iztacala, Universidad Nacional Autónoma de México, Avenida de los Barrios No. 1, Los Reyes Iztacala, 54090 Tlalnepantla, MEX, Mexico; ^2^Laboratorio de Inmunología Molecular, Facultad de Estudios Superiores Zaragoza, Universidad Nacional Autónoma de México, Batalla 5 de Mayo esquina Fuerte de Loreto, 09230 Iztapalapa, DF, Mexico

## Abstract

More than one-third of the world's population is infected with one or more helminthic parasites. Helminth infections are prevalent throughout tropical and subtropical regions where malaria pathogens are transmitted. Malaria is the most widespread and deadliest parasitic disease. The severity of the disease is strongly related to parasite density and the host's immune responses. Furthermore, coinfections between both parasites occur frequently. However, little is known regarding how concomitant infection with helminths and *Plasmodium* affects the host's immune response. Helminthic infections are frequently massive, chronic, and strong inductors of a Th2-type response. This implies that infection by such parasites could alter the host's susceptibility to subsequent infections by *Plasmodium*. There are a number of reports on the interactions between helminths and *Plasmodium*; in some, the burden of *Plasmodium* parasites increased, but others reported a reduction in the parasite. This review focuses on explaining many of these discrepancies regarding helminth-*Plasmodium* coinfections in terms of the effects that helminths have on the immune system. In particular, it focuses on helminth-induced immunosuppression and the effects of cytokines controlling polarization toward the Th1 or Th2 arms of the immune response.

## 1. Introduction

Currently, it is estimated that approximately one-third of the almost three billion people who live on less than two US dollars per day are infected with one or more helminths [1]. Human infections with these organisms remain prevalent in countries where the malaria parasite is also endemic [2]. Consequently, coinfections with both parasites occur frequently [3, 4]. These interactions could have potential fitness implications for both the host (morbidity and/or mortality) and the parasite (transmission). Several studies have shown that the ability of a parasite to successfully establish an infection will depend on the initial immune response of the exposed host [5, 6]. When entering the host, a parasite will experience an “immune environment” potentially determined by both previous and current infections [7–9]. It is widely recognized that, in the presence of Th2 effector response, Th1 response is suppressed and vice versa [10]. Thus, Th2-type response evoked in response to helminth infection would in theory have the ability to suppress proinflammatory Th1 response that generates immunopathology in* Plasmodium* infection.

Despite the fact that helminth parasites cause widespread, persistent human infection that results in a Th2 immune response, the influence of helminths on the duration of episodes of malaria in humans is not clear. The questions of how the coexistence of helminths and* Plasmodium* parasites within the same host might influence the immunological responses to each species and whether interactions affect resistance, susceptibility, and the clinical outcome of malaria has yet to be answered.

In this review, we attempt to answer these questions and particularly address whether the preexistence of a Th2/T regulatory response induced by helminths could affect the immune response against* Plasmodium*.

Before analyzing the influence of helminths infection on malaria, we must first briefly outline the immune response to* Plasmodium* infection and later outline the immune response to helminths parasites, as this is important to subsequent analyses of how malaria can be modified by the helminths.

## 2. *Plasmodium*

Malaria is caused by protozoan parasites belonging to the genus* Plasmodium*; it is transmitted by female* Anopheles* mosquitoes.* Plasmodium* is still one of the most successful pathogens in the world and is a major cause of morbidity and mortality in tropical countries. Five species of* Plasmodium* (i.e.,* P. falciparum*,* P. vivax*,* P. malariae*,* P. ovale*, and* P. knowlesi*) are responsible for all human infections [11, 12].

*Plasmodium* parasites have a complicated, multistage lifecycle involving an* Anopheline* mosquito vector and a vertebrate host. The parasite develops in two stages in its human host: in the liver (the exoerythrocytic stage) and in the blood (the intraerythrocytic stage). The most characteristic features of malaria in humans are a fever that occurs every 48 to 72 h depending on the species of* Plasmodium*, chills, headache, and gastrointestinal symptoms. In a naive, untreated individual, these can rapidly escalate into cerebral malaria (CM), anemia, severe organ failure, and death [13].

### 2.1. Immune Response during* Plasmodium* Infection

The immune response to* Plasmodium* is poorly understood; it depends on the parasite species and the specific stage within the host [12]. In addition, it is dichotomized into the preerythrocytic response, which is directed against the sporozoite and liver-stage parasites, and the blood stage response, which is directed against merozoites and intraerythrocytic parasites.

Although animal models do not fully replicate human malaria, they are invaluable tools for elucidating immune processes that can cause pathology and death [14]. Several mouse strains have been used to study the immune response to different combinations of* Plasmodium* species, such as* P. berghei* [15–20],* P. yoelii* [15, 21–24],* P. chabaudi* [25–30], and* P. vinckei* [31] (Table 1). These malarial models suggest that the efficiency of parasite control requires both a humoral and a cellular immune response, most likely in cooperation, although the importance of each is not entirely clear. For example, immunity to the sporozoite depends on antibodies to surface proteins, such as CSP-2 [32, 33] and liver-stage antigen (LSA-1) [34]; these antigens induce the production of antibodies that neutralize or block the invasion of hepatocytes [35]. Once sporozoites have entered the hepatocyte, the parasite clearance in mice requires CD8^+^ T cells [36], natural killer cells (NK), and NKT and *γ* *δ* T cells that produce IFN-*γ* to eliminate infected hepatocytes [35]. When the parasite invades red blood cells (RBC), it dramatically alters the physiological and biochemical processes of its host cell. Parasite-infected RBCs (pRBC) express parasite-encoded molecules on their surface that affects the RBCs' mobility and trafficking within the body. The parasite biomass increases very rapidly and activates innate immune mechanisms, including NK cells and *γ* *δ* T cells [13].

NK cells play an important role in restricting parasite replication. The absence of NK cells is associated with low IFN-*γ* serum levels and increased parasitemia in mice infected with* P. chabaudi* [37]. Likewise, the absence of IFN-*γ* reduces the ability of mice to control and eliminate parasites, eventually resulting in the death of the animals [38, 39]. Interestingly, macrophages (M*φ*), but not IFN-*γ*, play a major role in the control of early peaks in lethal infections with* P. yoelii* [40]. In addition, IFN-*γ* produced by CD4^+^ T cells plays a pivotal role in protective immunity against non-lethal strains of* Plasmodium* [41, 42]. In contrast, the infection with* P. berghei* ANKA induces high levels of IFN-*γ* and TNF-*α* which are associated with cerebral malaria [43]. However, the peak of parasitemia in athymic mice tends to be similar to the peak in WT mice. These results suggest that extrathymic T cells are the major lymphocyte subset associated with protection against malaria [44].

In a resistant strain of mice, the presence of the parasite induces the production of proinflammatory cytokines, such as IL-1*β*, IL-6, TNF-*α*, and IFN-*γ*. Furthermore, IL-12 is also necessary for elimination of* P. chabaudi* AS [45],* P. berghei* XAT [20], and* P. yoelii* XNL [46].

Besides, the inflammatory cytokine MIF (macrophage migration inhibitory factor) induces pathogenesis and susceptibility on BALB/c mice infected with* P. chabaudi*, high serum levels of MIF correlated with severity of disease [47]. In addition, infection of MIF knockout mice with* P. chabaudi* increases survival [48].

CD4^+^ T cells, together with B cells, are crucial to develop efficient protection in murine experimental models [49, 50]. Whereas IFN-*γ*, produced by TCD4+, activates M*φ*-mediated responses [51], the antibodies produced by B cells inhibit invasion of RBCs by the parasites [52], opsonize parasitized RBCs, block pRBC adhesion to the vascular endothelium, and neutralize parasite toxins [35]. In addition, mice rendered B cell deficient by treatment with anti-*μ* antibodies or B cell knockout mice (*μ*MT) are unable to clear the erythrocytic infection of* P. chabaudi* [50, 53, 54]. Specifically, the early acute infection is controlled to some extent, giving rise to chronic relapsing parasitemia that cannot be cleared. Finally, parasitemia can be reduced by adoptive transfer of B cells [50].

Antibodies also induce pathology due to parasite antigens that are freed and adhere to healthy erythrocytes; this generates anemia or autoimmune reactions that cause damage to the kidneys and other tissues [55–58]. For example, pathogenesis of malaria nephropathy is linked to subendothelial deposits of immune complexes containing IgG and IgM [59, 60]. The antibodies involved in the elimination of the parasite mainly belong to cytophilic subclasses (IgG1 and IgG3) [50, 61]. In addition, high levels of immunoglobulin E (IgE) correlate with protection against severe malaria [62–64].

Interestingly, after the peak of parasitemia, cellular immune responses should switch from Th1- to Th2-type response in* P. chabaudi* infected mice [65], because the malaria pathogenesis is caused by inappropriate or excessive inflammatory responses to eliminate the parasite [43, 66].

Interestingly,* Plasmodium* can modulate the response of antigen presenting cells, such as M*φ* and dendritic cells (DC), which leads to suppression of the immune response [67]. In the infection with* P. yoelii* YM, the DC function is affected by the presence of TNF-*α* [68]. Wykes et al. suggested that damage to the activity of DCs is due to a virulence factor that is present in certain parasite strains because, when DCs were transferred from mice infected with a “nonlethal” strain to mice infected with a “lethal” parasite strain, the mice were protected [46].

**Figure 1:**
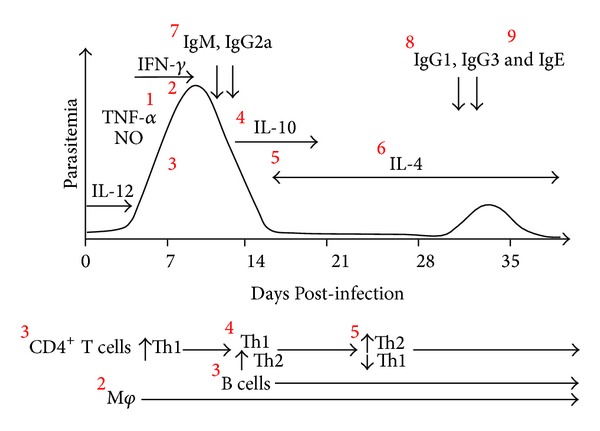
Representation of the course of* Plasmodium chabaudi* infection. Early infection with the erythrocytic stage is characterized by the production of proinflammatory cytokines, such as IL-12 and TNF-*α*, and a pronounced IFN-*γ* response. In addition, NO produced by M*φ* helped control parasitemia (1). IFN-*γ* activates M*φ*-mediated responses, in particular phagocytosis and elimination of pRBC (2). CD4^+^ T cells, together with B cells, are crucial for developing efficient protection (3). Th1 production is downregulated later by an increased Th2-type immune response following primary infection (4). In a later stage of infection, after the peak parasitemia has been reached, CD4 T cells switch from a Th1 to a Th2 cytokine profile (5). This switch helps B cells produce antibodies (6). The antibodies inhibit the invasion of RBCs by the parasites, opsonize parasitized RBCs, or block pRBC adhesion to the vascular endothelium (7, 8). The slow late switch from noncytophilic (IgM and IgG2a) (7) to cytophilic subclasses (i.e., IgG1 and IgG3) (8) is involved in parasite elimination (9). However, IgE correlates with protection against severe malaria. Figure modified from Langhorne et al. 2004 [75] and Stevenson and Urban 2006 [67].

The regulatory T cells are extremely important to control the inflammatory process in malaria, the number of CD4^+^ CD25^+^ Foxp3^+^ regulatory T cells (Treg) increases in mice infected with* P. yoelii* [69] or* P. berghei* [70]. In addition, mice infected with the lethal* P. yoelii* XL17 show higher levels of IL-10 and TGF-*β* compared to mice infected with the nonlethal strain* P. yoelii* XNL, at early time points during infection [71]. Furthermore, the suppression of T cells induces lethality in mice infected with* P. yoelii*, while neutralization of TGF-*β* and IL-10 decreases parasitemia and prolongs the survival of infected mice [71, 72]. Accordingly, Couper et al. reported that the main sources of IL-10 in lethal infection with* P. yoelii* are Treg cells [73]. Finally, the ablation of Treg cells from* P. yoelii*-infected DEREG-BALB/c mice significantly increases T cell activation and decreases parasitemia [74]. In addition, in mice infected with nonlethal strains of* P. yoelii*, the presence of cytokines such as IL-10 and TGF-*β* during the chronic phase of infection was detected [71]. Thus, these data together suggest that the outcome of malaria infection could be determined by the balance of proinflammatory and regulatory immune responses, which could inhibit pathology (Figure 1).

## 3. Helminths

Helminths are multicellular worms, some of which have adapted successfully to a parasitic lifestyle. They can be classified into three taxonomic groups: cestodes (e.g.,* Taenia solium*), nematodes (e.g.,* Ascaris lumbricoides*), and trematodes (e.g.,* Schistosoma mansoni*). Helminths vary in their biology in terms of size, lifecycle, and the diseases they cause. However, despite this complexity, helminths usually cause asymptomatic and chronic infections [76]. Helminths are among the most widespread infectious agents in human populations, especially in developing countries; they affect more than a third of the world's population, and more than 20 species infect humans (Table 2) [1, 77–83].

### 3.1. Immune Response during Helminth Infections

**Figure 2:**
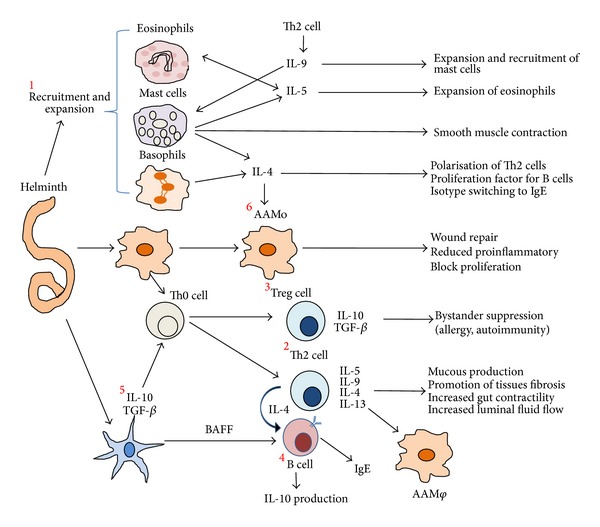
Helminth infections are strong inducers of a Th2-type immune response. These infections are characterized by the expansion and activation of eosinophils, basophils, and mast cells (1). Their upregulation due to high levels of immunoglobulin E (IgE) and the proliferation of T cells that secrete IL-4, IL-5, IL-9, and IL-13 are part of the host immune response against the parasite (2). However, helminth infections tend to be long-lived and largely asymptomatic because helminth infections are sustained through a parasite-induced immunomodulatory network, in particular through activation of regulatory T cells (3) and systemically elevated levels of IL-10 produced by B regulatory cells (4). They are additionally affected by the expression of the regulatory cytokines IL-10 and TGF-*β*, produced by regulatory dendritic cells (5) and alternatively activated M*φ* (AAM*φ*) (6).

Infection of mammals by helminth parasites typically results in a conserved series of immune events that are orchestrated and dominated by T helper cell type (Th2) events, characterized by the activation of eosinophils, basophils, and mast cells; high levels of immunoglobulin E (IgE); and the proliferation of T cells that secrete IL-4, IL-5, IL-9, and IL-13 [84, 85]. Despite this response, helminths are able to modulate and suppress the host immune response to promote their own survival and their persistence in the host for a long time, resulting in chronic infection [76, 86, 87]. These mechanisms include the ability to induce regulatory responses via regulatory T cells (Treg) which express molecules that inhibit the immune response, such as glucocorticoid-induced TNF-R-related protein (GITR) and the receptor cytotoxic T lymphocyte antigen 4 (CTLA-4) [88–91]. Treg cells also secrete suppressive cytokines, such as IL-10 and TGF-*β* [92]. On the other hand, B regulatory cells (Breg) also contribute to immune modulation and can release IL-10 and restrict proinflammatory responses [93]. Helminths also induce the differentiation of anti-inflammatory M*φ*, called alternatively activated M*φ* (AAM*φ*) [94, 95], as well as regulatory dendritic cells (DCreg), which are characterized by the expression of the regulatory cytokines IL-10 and TGF-*β* [96, 97] (Figure 2).

This anti-inflammatory or regulatory response could be potentially detrimental to the host if it interferes with the development of protection against other infections that require an inflammatory response, such as* Leishmania major* [9, 98] or* Trypanosoma cruzi *[8].

The hyporesponsive immune response induced during chronic helminth infection affects not only the response to helminth antigens but also to other antigens. Several studies have examined the effect of infections on the immune response to other unrelated antigens. In particular, it has been shown that the response to vaccines can be modified by the presence of concomitant helminth infection. For example, chronic* Onchocerca* infection [99],* Lymphatic filariasis* [42], or* Schistosoma* [100] reduces the effectiveness of the tetanus vaccine. Likewise, chronic* Onchocerca* infection affects* Bacillus Calmette-Guérin* and* Rubella* vaccinations [101]. Similarly,* Ascaris lumbricoides* reduces the response to the oral cholera vaccine, which can be restored by albendazole treatment [102]. However, helminthic infections are beneficial in the control of excessive inflammatory reactions, such as Crohn's disease [103] and ulcerative colitis [104], as well as in allergic diseases [105–107] and autoimmune diseases, such as encephalomyelitis [108, 109] and arthritis [110].

Despite the widespread acceptance that helminthic infections influence each other directly or indirectly, little attention has been paid to helminth-*Plasmodium* coinfections. One reason is that the interactions involved are complex and difficult to understand. Here, we will try to discuss several reports about helminth-malaria coinfections to clarify the consequences of this interaction.

## 4. Human* Plasmodium*-Helminth Coinfection

*Plasmodium* spp. infect between 349 and 552 million people and kill over one million each year; approximately 40% of the world's population is at risk of being infected [2, 111]. Importantly, people living in malaria-endemic regions are exposed to other pathogens, especially those associated with poverty, such as helminths.

Several studies have been carried out to explore the influence of helminths on* Plasmodium* infection in humans (Table 3). However, the evidences described in these researches are controversial. While some studies have reported that helminth infection favors protection because reduces the* Plasmodium* parasite density [112], promotes protection against clinical malaria [113, 114], reduces anemia [113, 115, 116], cerebral malaria [117] and renal failure [118] (Table 3(a)). Other studies showed no influence of helminths on the curse of* Plasmodium* infection [119–121] (Table 3(b)). In contrast, others showed an increased susceptibility to* Plasmodium* infection [114, 122], increased risk of complications [123–125], anemia [125, 126], hepatosplenomegaly [127, 128], and increased* Plasmodium* parasite load [129, 130] (Table 3(c)).

Although a Th2 phenotype is a conserved response to helminth infection in human and mice, the nature of the host immune response varies considerably between species of helminths; in some cases Th1 immune response predominates, depending on both the time of infection and the helminth development stage [131, 132]. The time that Th1 immune response is sustained until it polarizes toward Th2, could vary between species [133–135]. Thus, the controversial results related to helminth-*Plasmodium* coinfection in humans could be explained because many studies did not consider critical features of the helminth parasite biology. For example, the biological niche or parasite stage within the host. Neither the previous time of infection with the helminth nor the nutrition state and age of the host were taken into account.

Because all of these variables were not considered in existing studies in humans and in order to establish a possible consensus, we review in detail the murine* Plasmodium*-*helminth* coinfections, which in theory, controlled variables more rigorously.

## 5. Experimental Models of Coinfection

Although helminth infections in mice are a questionable model for chronic helminth infections in humans, the fact is that many intraintestinal helminths can reach large biomass which can change the cytokine environment and therefore the possible mechanisms of response. By establishing chronic infections and inducing strong Th2-type responses, helminths could have a potentially significant influence on the nature of the immune response in infected individuals and hence modify their susceptibility to subsequent infections with other important pathogens, at least those that require a Th1-type or mixed Th1-/Th2-type immune response, such as* Plasmodium* sp.

### 5.1. *Schistosoma-Plasmodium* Coinfection

According to the theory that Th2-type response evoked in response to helminth infection would have the ability to suppress proinflammatory Th1 response that generates immunopathology in* Plasmodium*-infected individuals, there are some reports of experimental models of coinfection with* Plasmodium berghei* ANKA (*Pb*) after* Schistosoma mansoni* (*Sm*) infection in ICR mice or with* Schistosoma japonicum*- (*Sj*-)* Pb* in C57BL/6 mice 7 or 8 weeks after helminthic infection, respectively; both coinfections showed a delay in death of mice [136, 137]. Interestingly, there was a reduction in the brain pathology associated with high levels of the anti-inflammatory cytokines IL-5, IL-10, and IL-13 [136–138].

In contrast, similar coinfection with* Pb* 7 or 8 weeks after* Sm* infection showed an increase in mortality and parasitemia in Swiss albino and C57BL/6 mice [138, 139]. Moreover, the coinfections in Swiss albino mice reduced the effectiveness of antimalarial treatments and delayed elimination of the parasite [139] (Table 4(a)). In these reports neither evidence of immune response nor pathology data were shown. Thus, we speculate that increase parasite load was probably due to the presence of helminth than inhibited Th1-type immune response which was able to contain the replication of* Plasmodium*, and the increased mortality was due to parasite load rather than a pathological Th1-state dependent. In line with this hypothesis, coinfection with* Plasmodium chabaudi* (*Pc*) at 8 weeks after* Sm* infection in C57BL/6 mice allowed high* Pc* replication. This increase was associated with low levels of the proinflammatory TNF-*α* [140].

It is known that the immune response against* Schistosoma* shifts from an early helminth-protective Th1-type immune response to a late helminth-permissive Th2-type response during the course of infection [134]. Thus, the moment when the second infection is acquired (2, 4 and 6 weeks post-helminth infection) would be critical for disease outcome and pathology [141]. These findings could be supported by the fact that chronically* Sm*-infected BALB/c mice coinfected (6 weeks) with the nonlethal strain* Plasmodium yoelii* NXL (*Py*NXL) showed high mortality. In contrast, no mortality was observed in acutely (2 or 4 weeks) coinfected mice, although they developed high parasitemia and hepatomegaly was higher in coinfected mice compared with mice infected with each parasite separately [141] (Table 3(a)). Therefore, the time of previous infection may influence the response against* Plasmodium*.

Together, these reports suggested that the Th2 response, induced by* Schistosoma*, plays an important role in protecting against immunopathology in cerebral malaria. However, the presence of* Schistosoma* does not appear to modify the virulence of* Pb* and, consequently, it does not alter the lethality of* Plasmodium* infection.

Finally, one report scape to the theory that Th2-type immune response evoked by the helminth infection would possess the ability to suppress the proinflammatory Th1-type response in its host.* Sm*-infected A/J mice coinfected at 8 weeks with* Pc* were protected by the presence of concomitant* Sm* infection. The mice escaped death due to malaria; this effect was accompanied by enhanced levels of IFN-*γ* [142] (Table 4(a)).

### 5.2. *Heligmosomoides polygyrus-Plasmodium* Coinfection

Several studies used mice of the same genetic background. Additionally, equivalent helminth and* Plasmodium* strains have been used to explain whether previous helminthic infection plays an important role in the immune response against* Plasmodium*. Su et al. showed that C57BL/6 mice previously infected with* Heligmosomoides polygyrus* (*Hp*) and challenged with* Pc* either 3 or 5 weeks after helminthic infections developed high* Pc*-parasitemia and mortality, which was associated with low levels of IFN-*γ* and high levels of TGF-*β* and IL-10 [143]. However,* Hp-Pc* coinfection at 2 weeks resulted in less severe pathology (i.e., less hypothermia and hypoglycemia) and induced earlier reticulocytosis compared with mice infected only with* Pc* [144] (Table 4(b)).

Helmby in 2009 showed that mice developed high mortality in the* Hp-Pc* model when the two infections were introduced simultaneously. The mortality was due to severe liver pathology associated with increased IFN-*γ*, IL-17, and IL-22. Interestingly, when using an IFN-*γ* and IL-23 knockout strain, the mice survived the coinfection [145]. Thus, simultaneous* Hp*-*Pc* coinfection increased mortality, which may be a consequence of a synergistic effect that increased the inflammatory response (Table 4(b)).

In fact, in the first case, in which* Hp-Pc* coinfection was performed at 3 or 5 weeks after the initial helminthic infection, the high mortality observed may have been due to the anti-inflammatory response generated by the previous helminthic infection, which inhibited the inflammatory response necessary for control of the* Plasmodium* infection. However, when the coinfection was performed at the same time, mice developed a stronger inflammatory response, which generated greater pathology and mortality. This susceptibility is supported by the observation that chronic helminthic infection suppresses effective vaccine-induced protection against* Plasmodium.* However, when mice were administered with antihelminthic* Hp* treatment before malaria vaccination, the protective immunity against* Pc* was restored [146]. Therefore, the timing of the infection with* Hp* plays an important role in the type of immune response that is generated within the host, and it determines the susceptibility following challenge with* Plasmodium*.

The genetic background of mice infected with helminths has a crucial role in the outcome of the immune response to* Plasmodium*. For example, coinfection with the nonlethal* Py*NXL strain at 2 weeks after* Hp* infection in C57BL/6 mice resulted in exacerbated pathology and poor survival of mice. This susceptibility was associated with a reduced response against* Py*NXL (i.e., low levels of IFN-*γ*) in the spleen cells. As a consequence, it increased the activation of Treg cells [147]. However, the same coinfection at 3 weeks in BALB/c mice decreased the pathology associated with low levels of IFN-*γ* and increased levels of IL-4, but not IL-10 [148]. Therefore, the genetic background of mice infected with the helminth determines the outcome of* Py*NXL infection (Table 4(b)).

What happens when a lethal strain of* Plasmodium* was used in coinfection with* Hp*? The* Pb* ANKA infection in C57BL/6 mice induced typical symptoms of ECM [160, 161]. Coinfection with* Hp-Pb* ANKA 2 weeks after initial helminthic infection did not modify the development of ECM despite accelerated* Pb* growth* in vivo* [149]. Likewise, other results from the same model of coinfection in BALB/c and C57BL/6 mice showed no differences in parasitemia, anemia, or body weight in relation to mice infected only with* Plasmodium* [150]. Therefore,* Hp* infection does not affect the outcome of* Pb* ANKA (Table 4(b)).

### 5.3. *Echinostoma caproni*-*Plasmodium* Coinfection

Studies in BALB/c mice infected for 3 weeks with* E. caproni* (*Ec*) and then coinfected with the nonlethal strain* Py*NXL showed that exacerbation of* Plasmodium*-induced pathology was associated with a deficit in IFN-*γ* production [148]. Similarly, when* Ec*-infected mice were coinfected at 5 weeks, increased mortality was observed. The exacerbated pathology was reversible through the clearance of* Ec* worms via praziquantel treatment [151]. However, coinfection at 5 weeks with the lethal* Py*XL strain did not alter the course of infection; all mice infected with* Py*XL (i.e., alone, in combination with* E. caproni*, or praziquantel treated) died on day 10 after infection [151] (Table 4(c)). Therefore,* Ec* infection does not affect the outcome of lethal* Py*XL, but* Ec* infection affects the protective response against a nonlethal* Plasmodium* strain.

### 5.4. *Strongyloides ratti*-*Plasmodium* Coinfection

Murine* Strongyloides ratti* (*Sr*) infection is a transient helminthic infection that is resolved spontaneously within 3-4 weeks. This infection induces a strong Th2-type immune response at day 6 after infection [135]. When BALB/c mice were coinfected with the nonlethal strain* Py*NXL at day 6 after* Sr* infection,* Sr* induced a slightly enhanced peak of* Plasmodium* parasitemia and loss of body weight. In contrast, in C57BL/6 mice coinfected at day 6, parasitemia level and body weight were not altered. Interestingly, the Th2-type immune response induced by* Sr* was significantly reduced upon* Py*NXL coinfection [152]. In addition,* Py*NXL clearance was not affected by previous infection with* Sr* in either C57BL/6 or BALB/c mice. Moreover, infection with* Sr* in BALB/c mice did not change the efficacy of vaccination against* Pb* ANKA [153]. Therefore, infection with* Sr* does not affect the protective response against* Plasmodium*, although it generates small changes in parasitemia levels; which is not decisive for the outcome of* Plasmodium* infection (Table 4(d)).

### 5.5. *Nippostrongylus brasiliensis-Plasmodium* Coinfection

BALB/c mice infected with* Nippostrongylus brasiliensis* (*Nb*) exhibit a strong Th2-type immune response [162]. Even so, when BALB/c mice were coinfected with* Nb* and* Pc* simultaneously, the Th2 response against* Nb* was impaired by* Plasmodium*. Interestingly, the* Nb-Pc* coinfection had a beneficial effect; it slightly ameliorated the severity of malarial anemia (SMA) and decreased parasitemia levels [154]. Similarly, C57BL/6 mice infected for 3 weeks with* Nb* and then coinfected with* Pb* showed a delayed peak parasitemia and an increased survival time [155]. Thus, the presence of concomitant* Nb* infection plays an important role in inhibiting pathology associated with a challenge with* Pc* or* Pb* (Table 4(e)).

### 5.6. Coinfection with Other Helminths

Experimental models of coinfection with* Litomosoides sigmodontis* (*Ls*) 8 weeks and* Pc* infection in BALB/c mice showed increased SMA and weight loss associated with increased levels of IFN-*γ* [156]. In contrast, coinfection with* Ls* 8 weeks and* Pb* infection in C57BL/6 mice showed significantly reduced ECM rates associated with increased levels of IL-10. This protection was inhibited in IL-10 KO mice [157]. High levels of IL-10 were important in reducing pathology but also interfered with the protective response to* Plasmodium* in the liver. In particular, chronic infection with* Ls* interfered with the protective efficacy of a vaccine against sporozoite* Pb* in the liver [153]. Therefore, infection with* Ls* exacerbates the pathology of a* Pc* infection. In contrast, infection with* Ls* inhibits pathology in* Pb* infection due to an anti-inflammatory cytokine response (Table 4(f)).

In addition, CBA/J mice infected with* Brugia pahangi* (*Bp*) for 1 week and then coinfected with* Pb* displayed a low mortality rate, and mice were protected against the development of ECM. This protection was associated with increased serum IgE levels and Th2 cytokine production [158] (Table 4(g)). Similarly, infection with* Trichinella spiralis* (*Ts*) for 1 or 4 weeks in C57BL/6 mice greatly enhanced their resistance against the fatal coinfection with* Pb* [159]. Therefore, these observations suggest that the Th2-type immune response reduces brain pathology and increases survival in* Bp-* or* Ts*-*Pb* coinfection, perhaps due to the anti-inflammatory environment generated by the previous helminth infection (Table 4(h)).

The studies described above lead to different conclusions, while some of them suggest that prior infection with helminths induces resistance to* Plasmodium* [136, 137, 142, 148, 154, 155, 157–159], other studies do not show effects [149–153] and finally some others demonstrated an increased susceptibility to* Plasmodium* infection [138–141, 143–148, 151, 153, 156]. These contrasting results may partially be explained because this interaction is affected by the timing between the hosts' exposure to the helminth and* Plasmodium*. In addition, the strain of each parasite is also important, coinfection with nonlethal* Plasmodium* strains in the early stages of a helminthic infection delayed the onset of parasitemia due to early, specific high production of IFN-*γ*, but this response increased pathology. In contrast, a significant increase in susceptibility to nonlethal* Plasmodium* was observed when mice were coinfected with* Plasmodium* in the late stages of helminthic infection, when the Th2-type immune response is predominant.

Coinfection with lethal strains of* Plasmodium* in the late stages of a helminthic infection inhibits severe pathology and increases the survival of mice due to a decrease inflammatory response (mainly IFN-*γ* and TNF-*α*). In addition, the presence of a late anti-inflammatory Th2-type immune response induced by helminthic infection extended the survival of mice susceptible to* Plasmodium* infection; this may be due to a reduced pathological Th1-type immune response or may be due to induction of protective mix of Th1 and Th2 immune response. Recruitment and activation of M*φ* are essential for the clearance of malaria infections, but these have also been associated with adverse clinical outcomes [163]. Specifically, immunopathology of severe malaria is often originated from an excessive inflammatory Th1-type immune response. The expansion of Treg cells and the alternative activation of M*φ* by helminth infections may modulate the excessive inflammatory response to* Plasmodium*. Therefore, the chronic helminth infections inhibited pathology and increased survival in the challenge with lethal strains of* Plasmodium*.

## 6. Conclusions

The findings in this review demonstrate that the immune environment generated by a previous helminthic infection influences the response against* Plasmodium*. A helminth that persists in its host is able to significantly modify the host's susceptibility to or protection from* Plasmodium*. These modifications are dependent on the genetic background of mice, the type of helminth, and the time-course of the initial helminthic infection, which is crucial to the resulting immune response to* Plasmodium*.

**Figure 3:**
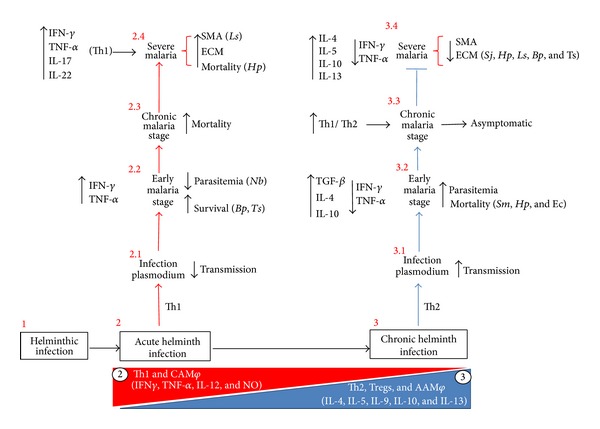
Concomitant helminth infection modified the immune response and susceptibility to* Plasmodium* infection. Helminth parasites have developed complicated strategies to infect and successfully colonize their host. (1) In an acute helminth infection, an initial Th1-like immune response (i.e., IFN-*γ*, IL-12, and classical activation macrophage (CAM*φ*)) is associated with low parasite growth. (2) However, as the parasite colonizes the host, the immune response rapidly shifts toward a Th2-dominant response (IL-4, IL-5, IL-10, IL-13, and AAM*φ*) in parallel with increased helminth parasitemia. (3) This “immune environment” determined by helminth infection modifies the immune response and the susceptibility to* Plasmodium.* That is, acutely helminth-infected mice exhibited (2) decreased transmission of* Plasmodium* (2.1), decreased parasitemia and increased survival (2.2) due to high levels of IFN-*γ* and TNF-*α* in the early stage. However, this immune response increased mortality during the chronic stage of malaria (2.3) and increased severe pathology, such as ECM and severe malaria anemia (SMA) (2.4). In contrast, chronically helminth-infected mice (3) increased the transmission of* Plasmodium* (3.1), parasitemia and mortality (3.2) due to high levels of IL-4, IL-10, and TGF-*β* and low levels of IFN-*γ* and TNF-*α*. However, during the course of the coinfection, the Th1 response against* Plasmodium* was increased. In fact, a mixed Th1/Th2 response during the chronic stage induced low levels of parasitemia and was asymptomatic (3.3). Interestingly, chronic helminth infections inhibited severe pathologies caused by* Plasmodium*, such as ECM and SMA (3.4), and increased the survival due to a decreased inflammatory response. Abbreviations:* Schistosoma mansoni* (*Sm*),* Heligmosomoides polygyrus* (*Hp*),* Echinostoma caproni* (*Ec*),* Strongyloides ratti* (*Sr*),* Nippostrongylus brasiliensis* (*Nb*),* Litomosoides sigmodontis* (*Ls*),* Brugia pahangi* (*Bp*), and* Trichinella spiralis* (*Ts*).

The impact of helminth-*Plasmodium* coinfection on acute helminthic infection increased or synergized the Th1-type immune response. This might be successful in inducing a response that inhibits* Plasmodium* replication, but it increases the pathology and mortality in the host. Alternatively, chronically helminth-infected mice showed a shift toward Th2-type immune responses. This could render the host more susceptible to* Plasmodium* infection and favor their replication; however, this response protected the host from severe malaria (Figure 3). Overall, these results suggest that malarial immunity is influenced by helminth infections. Therefore, the study and manipulation of antimalarial immunity seems difficult in the absence of any information concerning the effects of helminths on this response.

## 7. Perspectives

The helminth-*Plasmodium* interaction may have undesirable implications for global public health; for example, malaria vaccines trials do not consider the immune response to helminths, and this could result in decreased performance or cause adverse effects. Thus, a better understanding of helminth-induced regulation in the antimalarial response is indispensable for the rational development of effective antimalarial vaccines and novel therapies to alleviate or prevent the symptoms of severe malaria. The risk that entire populations may have an increased susceptibility to* Plasmodium* should invite study regarding the possible epidemiological relevance of helminth infections and the impact of controlling them on malaria incidence. The presence of helminth infections could represent a much more important challenge for public health than previously recognized. Therefore, we would emphasize that it is extremely important to carry out experiments in animal models that use more rigorous criteria to define exhaustively all the ramifications of immune regulation and potential side effects of helminth infection in the context of malaria. These results would allow extrapolate the observation in human populations presenting malaria.

## Figures and Tables

**Table 1:** Mouse models of malaria infection. ECM: experimental cerebral malaria, PvAS: *P. vinckei* petteri arteether sensitive, PvAR: *P. vinckei* arteether resistant, *Py*: *P. yoelii*, and KO: knockout.

Species	Subspecies: clone	Mouse strain and anemia	Mouse strain and CM	Useful in research	Ref.
*P. berghei *	*P. berghei ANKA *	C57BL/6: lethal CD-1: lethal C57BL/6J: non-lethal BALB/c: lethal	C57BL/6: susceptible CBA: susceptible BALB/c: resistant	Used as a model of ECM; there is genetic variation in the development of ECM between inbred strains	[15–17]
	*P. berghei K173 *	C57BL/6: lethal		Used to study pathogenesis; differs in some aspects of pathogenesis, indicating the influence of parasite genetic variation	[18]
	*P. berghei NK65 *	C57BL/6: lethal		Is a murine noncerebral malaria strain; induces a progressive increase in parasitemia, intense hepatic inflammation, and death	[19]
	*P. berghei XAT *	Spontaneously cleared in immune competent mice		Irradiation-induced attenuated variant from lethal strain *Pb* NK65; comparison of immune responses induced by these lethal and attenuated parasites lead us to elucidate the mechanisms of protective immunity and pathogenesis	[20]

*P. yoelii *	*P. yoelii 17 NXL *	BALB/c: non-lethal	Most strains resistant	Used to study immune mechanisms and pathogenesis; *Py*; line A1 is a mild line which is restricted to reticulocytes	[15]
	*P. yoelii 17XL *	BALB/c: lethal C57BL/6: lethal	Most strains susceptible	Used to identify vaccine-induced immune response	[21, 22]
	*P. yoelii *YM	CBA: lethal		*Py*-YM is virulent infection which multiplies in both immature and mature erythrocytes	[23]
	*P. yoelii *YA	CBA: non-lethal		YM parasites are responsible for normocyte invasion, increased virulence compared to mild line *Py* YA parasites; lines YM and A/C differed additionally in enzyme and drug-sensitivity markers	[24]

*P. chabaudi *	*P. chabaudi AS *	A/J: lethal C57BL/6: non-lethal BALB/c: non-lethal	C57BL/6 IL-10KO: susceptible	Used to study immune mechanisms and immunoregulation by cytokines, to identify susceptibility loci, and to study the immune basis of pathology	[25–28]
	*P. chabaudi AJ *	BALB/c: non-lethal		Used to study experimental vaccines and immunological processes that control hyperparasitaemia	[25, 27]
	*P. chabaudi* *adami DS *	C3H: lethal C57BL/6: non-lethal		Is fast-growing and high pathogenicity, induces more anemia, weight loss, and is less infective to mosquitoes than DK strain	[29, 30]
	*P. chabaudi* *adami DK *	BALB/c: non-lethal C3H: non-lethal		Is slower growing and less pathogenic and more selective in its invasion of subset of RBCs than DK	[29, 30]

*P. vinckei *	*P. vinckei vinckei *	BALB/c: lethal AKR: lethal		Used to study pathogenesis and for chemotherapy studies; it causes aggressive, overwhelming hyperparasitaemia	[31]
	*P. vinckei petteri *	AKR: lethal (PvAS) AKR: non-lethal (PvAR)		Used for drug screening and immunological studies	[31]

**Table 2:** Prevalence of common helminths in the world. These are estimates of the number of people with active infections. The number of people potentially exposed or with subclinical helminthic infections is much higher.

	Helminth	Estimated number of infected people	Ref.
Nematodes	*Ascaris lumbricoides *	1450 billion	[77]
	*Trichuris trichiura *	1050 million	[77]
	*Ancylostoma duodenale *	740 million	[78]
	*Trichinella spiralis *	600 million	
	*Necator americanus *	576 million	[1]
	*Brugia malayi *	157 million	[1]
	*Wuchereria bancroftiand Brugia malayi *	120 million	[79]
	*Strongyloides stercoralis *	100 million	[80]
	*Onchocerca volvulus *	37 million	[81]
	*Loa loa *	13 million	[1]

Trematodes	*Schistosoma spp. *	207 million	[82]
	*Fasciola hepatica *	17 million	

Cestodes	*Taenia spp. *	0.4 million	[81]
	*Hymenolepis nana *	75 million	
	*Echinococcus spp. *	2–3.6 million	[83]

**(a):** 

Study area	Age of group	Sample (size)	Study design	Helminth type	Outcome for malaria diseases in coinfection	Ref.
Senegal (Niakhar)	Children	178	Over a 2-year followup period	*S. haematobium *	Children with a light *S. haematobium* infection presented lower *P. falciparum* parasite densities than children not infected by *S. haematobium *	[112]
Mali (Tieneguebougou and Bougoudiana)	Children and young adults	62	Followed prospectively through a malaria transmission season	*Wuchereria bancrofti* *Mansonella perstans *	Pre-existent filarial infection attenuates immune responses associated with severe malaria and protects against anemia, but has little effect on susceptibility to or severity of acute malaria infection	[113]
Southern Ethiopia	1 to 82 years Mean 18.6 years	1,065 febrile patients	Cross-sectional	*A. lumbricoides* *T.trichiura, S. mansoni,* and hookworm	The chance of developing non-severe malaria were 2.6–3.3 times higher in individuals infected with helminth, compared to intestinal helminth-free individuals The odds ratio for being infected with non-severe *P. falciparum* increased with the number of intestinal helminth species	[114]
South-central Côte d'Ivoire	Infants (6–23 months), children (6–8 year), and young women (15–25 years)	732 subjects	Cross-sectional survey	Soil-transmitted helminth	Coinfected children had lower odds of anemia and iron deficiency. Interaction between *P. falciparum* and light-intensity hookworm infections vary with age.	[115]
Brasil (Careiro)	School children 5 to 14 years	236	Cohort and cross-sectional	*A. lumbricoides* hookworm and *T. trichiura *	Helminthes protect against hemoglobin decrease during an acute malarial attack by *Plasmodium. *	[116]
Thailand (Bangkok)	Mean 24 years (range 15–62)	537 files	*Retrospective * case-control	*A. lumbricoides *	Percentage protection for mild controls against cerebral malaria ranged from 40% for *Ascaris *(present/absent) to 70% for *Ascaris *medium infection. For intermediate controls protection against cerebral malaria was 75% for *Ascaris *(present/absent).	[117]
Thailand (Bangkok)	19–37 years 22 patients with malaria-associated ARF and 157 patients with MSM	179	*Retrospective* case-control	*A. lumbricoides, T. trichiura, *hookworm, and *Strongyloides* *stercoralis *	Helminths were associated with protection from renal failure Helminth-infected controls were less likely to have jaundice or to have peripheral mature schizonts than controls without helminths	[118]

**(b):** 

Study area	Age of group	Sample (size)	Study design	Helminth type	Outcome for malaria diseases in coinfection	Ref.
Kenya (Kingwede)	8 years and older	561	cross-sectional	*S. haematobium *	Children had 9.3 times the odds of coinfection compared to adults	[119]
Nigeria (Osun)	preschool children (6–59 months)	690	Double-blind and randomized	*A. lumbricoides *	There was no significant difference in the severity of anaemia.	[120]
Kabale, Uganda	All ages (856)	856	Retrospective; 18 months	*A. lumbricoides, T. trichiura,* and hookworm	Non evidence for an association and risk of malaria	[121]

**(c):** 

Study area	Age of group	Sample (size)	Study design	Helminth type	Outcome for malaria diseases in coinfection	Ref.
Senegal (Niakhar and Bambey)	Children, mean 6.6 years	105	Prospective case-control	*A. lumbricoides *	Prevalence of *A. lumbricoides *infection was higher in cases of severe malaria	[123]
Northern Senegal	Children aged 6–15 years	512	Cohort	*S. mansoni *	The incidence rate of malaria attacks was higher among *S. mansoni*-infected individuals carrying the highest worm loads. In contrast, the rate of malaria attacks were lower in medium grade *S. mansoni *infections	[124]
Ghana (Kumasi)	Women (15–48 years) mean 26.8 years	746	Cross-sectional	*A. lumbricoides, T. trichiura, S. stercoralis,* and *E. vermicularis *	Coinfection resulted in increased risks of anemia, low birth weight, and small for gestational age infants	[125]
Ethiopia (Alaba Kulito)	Children <5 years, children 5–14 years, and adults ≥15 years	1802 acute febrile patients	case-control	Hookworm, *A. lumbricoides*, and *T. trichiura *	Coinfection is associated with higher anaemia prevalence and low weight status than single infection with* Plasmodium *in children	[126]
Kenya (Makueni)	Primary school children 4–17 years	(221 and 228)	Cross-sectional	*S. mansoni *	Hepatosplenomegaly due to proinflammatory mechanism exacerbated by schistosomiasis	[127]
Kenya (Mangalete)	Children 4–17 years	79	Cross-sectional	*S. mansoni *	Hepatosplenomegaly is associated with low regulatory and Th2 response to *Schistosome* antigens	[128]
Zimbabwe (Burma Valley)	Children 6–17 years	605	12-month followup of a cohort of children	Schistosome	Increased prevalence of malaria parasites and had higher sexual stage malaria parasite in children coinfected with schistosomiasis	[129]
Cameroon (Bolifamba)	9 months to 14 years	425 children		*A. lumbricoides*, *T. trichiura*, and hookworm	Coinfections in which heavy helminth loads showed high *P. falciparum *parasite loads compared with coinfections involving light helminth burden	[130]

**(a):** 

Background mouse	*Plasmodium* strain	Helminth type	Coinfection time*	Malaria disease outcome	Ref.
ICR HSD	*P. berghei* ANKA	*S. mansoni *	7 wks	Low rates of ECM (30%), delay in death associated with high levels of IL-4, IL-10	[136]
C57BL/6	*P. berghei* ANKA	*S. japonicum *	8 wks	Increased survival rate and reduction of the brain pathology. Th2 response induced by worm plays an important role in protecting against ECM	[137]
C57BL/6	*P. berghei* ANKA	*S. mansoni *	8-9 wks	Increased parasitemia, mortality, weight loss, and hypothermia; decreased pathology in the brain associated with high levels of IL-5, IL-13 and low serum IFN-*γ*	[138]
Swiss albino	*P. berghei* ANKA	*S. mansoni *	7 wks	Increased parasitemia and mortality Delayed reduction/elimination of the parasite followed by administration of antimalarial treatment	[139]
C57BL/6	*P. chabaudi*	*S. mansoni *	8 wks	Increased parasitemia associated with a deficiency in the production of TNF-*α*	[140]
BALB/c	*P. yoelii* NXL (non-lethal)	*S. mansoni *	2, 4, and 6 wks	Increased parasitemia and death at 6 wks of coinfection. Hepatosplenomegaly was more marked in coinfected mice compared to either disease separately	[141]
A/J	*P. chabaudi *	*S. mansoni *	8 wks	Mice escape death and showed high production of IFN-*γ*	[142]

**(b):** 

Background mouse	*Plasmodium* strain	Helminth type	Coinfection time*	Malaria disease outcome	Ref.
C57BL/6	*P. chabaudi *	*H. polygyrus *	2, 3, or 5 wks	Increased parasitemia and mortality associated with low levels of IFN-*γ* and high levels of TGF-*β*, IL-10	[143]
C57BL/6	*P. chabaudi AS *	*H. polygyrus *	2 wks	Increased parasitemia; however, it ameliorates severe hypothermia and hypoglycaemia; besides this, it induced earlier reticulocytosis than *Pc*-infected WT mice	[144]
C57BL/6 IFN^−^/_−_ IL-23^−^/_−_	*P. chabaudi AS *	*H. polygyrus *	At the same time	Increased mortality and severe liver disease, associated with increased IFN-*γ*, IL-17, and IL-22 in the liver. The coinfected IFN^−^/_−_ and IL-23^−^/_−_ mice survive	[145]
C57BL/6 BALB/c	*P. chabaudi AS *	*H. polygyrus *	2 wks with AgPc + adjuvant	Suppresses the protective efficacy of the malaria vaccine. Deworming treatment before antimalarial immunization restored the protective immunity to malaria challenge	[146]
C57BL/6	*P. yoelii* 17 XNL	*H. polygyrus *	2 wks	Increased pathology due to reduced response against *Py* (low levels of IFN-*γ*) in the spleen cells, as a result of higher activation of Treg	[147]
BALB/c	*P. yoelii* 17 NXL	*H. polygyrus *	3 wks	Reduction of pathology, low levels of IFN-*γ*, and high levels of IL-4 induced by helminthes	[148]
C57BL/6	*P. berghei* ANKA	*H. polygyrus *	2 wks	*Hp* infection did not alter ECM development, despite accelerated *P. berghei* growth *in vivo *	[149]
C57BL/6 BALB/c	*P. berghei* ANKA	*H. polygyrus *	2 wks	No differences	[150]

**(c):** 

Background mouse	*Plasmodium* strain	Helminth type	Coinfection time*	Malaria disease outcome	Ref.
BALB/c	*P. yoelii* 17 NXL	*E. caproni *	3 wks	*Ec* showed counterregulatory antiparasite cytokine responses to non-lethal strain *Py*NXL (less IFN-*γ* and high IL-4 levels induced by *Ec*)	[148]
BALB/c	*P. yoelii* 17 NXL	*E. caproni *	5 wks	Increased mortality and pathology; the pathology was reversible through clearance of *Ec* by praziquantel treatment	[151]
BALB/c	*P. yoelii* 17XL	*E. caproni *	5 wks	*Ec* does not alter the course of *Py*17XL infection	[151]

**(d):** 

Background mouse	*Plasmodium* strain	Helminth type	Coinfection time*	Malaria disease outcome	Ref.
C57BL/6	*P. yoelii* 17NXL	*Strongyloides ratti *	1 wk	Did not altered cytokine response	[152]
BALB/c	*P. berghei* ANKA	*Strongyloides ratti *	1 wk	The coinfection did not change the efficacy of vaccination against *Pb *	[153]

**(e):** 

Background mouse	*Plasmodium* strain	Helminth type	Coinfection time*	Malaria disease outcome	Ref.
BALB/c	*P. chabaudi *	*Nippostrongylus* *brasiliensis *	Same day	Reduction of anemia and parasitemia. Th2 response was inhibited by *Plasmodium *	[154]
C57BL/6	*P. berghei *	*Nippostrongylus brasiliensis *	3 wks	Delayed peak parasitemia, increased survival	[155]

**(f):** 

Background mouse	*Plasmodium* strain	Helminth type	Coinfection time*	Malaria disease outcome	Ref.
BALB/c	*P. chabaudi *	*L. sigmodontis *	8 wks	Increased severity of the anemia and weight loss associated with increased IFN-*γ*	[156]
C57BL/6 IL-10KO	*P. berghei* (ANKA)	*L. sigmodontis *	8 wks	Reduction of ECM associated with increased IL-10 IL-10KO mice coinfected with *Pb*-*Ls* die of ECM	[157]
BALB/c	*P. berghei* ANKA	*L. sigmodontis *	2 wks	Reduced protection against *P. berghei* challenge infection for low frequencies of CSP-specific CD8 T cells, CSP-specific IFN-*γ* and TNF-*α* production	[153]

**(g):** 

Background mouse	*Plasmodium* strain	Helminth type	Coinfection time*	Malaria disease outcome	Ref.
CBA	*P. berghei* (ANKA)	*Brugia pahangi* irradiated attenuated	1 wk	Increased survival and protected them against the ECM development; increase synthesis of IFN-*γ*, IL-4, IL-5, and IgE	[158]

**(h):** 

Background mouse	*Plasmodium* strain	Helminth type	Coinfection time*	Malaria disease outcome	Ref.
C57BL/6	*P. berghei *	*Trichinella spiralis *	1–4 wks	Partially subdued parasitaemia and prolonged survival	[159]
